# Integrate and conquer: pan-cancer proteogenomics uncovers cancer vulnerabilities and therapeutic opportunities

**DOI:** 10.1038/s41392-024-02009-6

**Published:** 2024-10-15

**Authors:** Debomita Chakraborty, Rossana Romero, Krishnaraj Rajalingam

**Affiliations:** Cell Biology Unit, University Medical Center Mainz, JGU-Mainz, Mainz, Germany

**Keywords:** Cancer genomics, Tumour heterogeneity

In a recent article published in *Cell* by Savage et al., the authors developed a computational workflow for integrating multi-omics data from readily available public online databases to provide novel insights into the proteogenomic landscape of several types of cancers and reveal new druggable targets for drug development or repurposing.^1^

Even though cancer causes over ten million deaths annually worldwide, only a few proteins are currently targeted by cancer drugs approved by the Food and Drug Administration. Most patients still receive traditional therapies including surgery, chemotherapy, and radiotherapy, which are often linked to recurrence, resistance, mutations, and toxicity. Proteogenomics, which combines genomics, transcriptomics, proteomics as well as phosphoproteomics data from tumors, holds great promise for enhancing our understanding of cancer mechanisms from genomic to physiological levels, and forms an important foundation for developing novel rational therapy approaches and advancing drug research. The National Cancer Institute’s Clinical Proteomic Tumor Analysis Consortium (CPTAC) provides a rich, coherent and powerful proteogenomics dataset generated by harmonizing multi-omic analyses as well as clinical data from more than one thousand tumors from ten different cancer types, including glioblastoma (GBM), head and neck squamous cell carcinoma (HNSCC), lung adenocarcinoma (LUAD), lung squamous cell carcinoma (LSCC), breast cancer (BRCA), pancreatic ductal adenocarcinoma (PDAC), clear cell renal cell carcinoma (ccRCC), high-grade serous ovarian cancer (HGSC), uterine corpus endometrial carcinoma (UCEC), and colorectal adenocarcinoma (COAD). However, computational analysis of individual and integrated cancer cohorts from such vast multi-omics resources across cancer subtypes is a challenging task.^2^

**Fig. 1:**
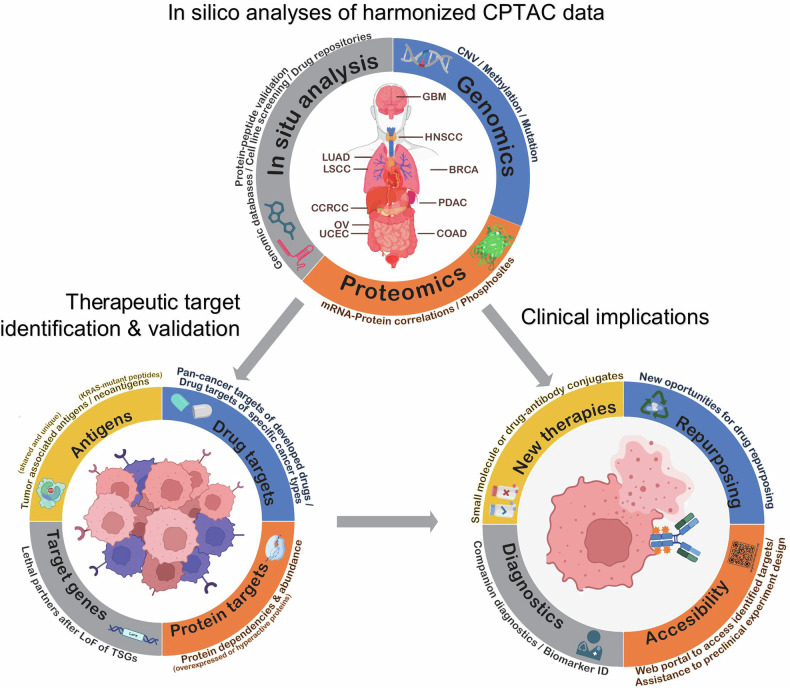
Computational workflow for data integration of public databases to identify new drug targets. Top: Computational process of data integration between CPTAC data and public online genomic and cell line databases, drug repositories and online resources for protein and peptide validation. (CNV: cell number variation) Lower left: Comparison of normal and tumor samples led to the identification and validation of partner genes after loss of function (LoF) of tumor suppressor genes (TSGs), protein dependencies that led to loss of fitness in cancer cell lines, KRAS mutants neoantigens and tumor associated antigens that show highly restricted expression in normal tissues. Lower right: Potential clinical implications after this study include drug repurposing, development of new small-molecules drugs or drug-antibody conjugates, novel therapies for companion diagnostics and access to a user-friendly web portal for the identified targets. Partially created with BioRender.com and Adobe Illustrator. Adapted from Savage, S.R. et al.^1^

Savage et al. took up this challenge and performed comprehensive proteogenomic analyses integrating the vast CPTAC pan-tumor data with several other public datasets with the aim to identify overexpressed or hyperactivated targetable protein dependencies, tumor suppressor gene (TSG) loss-associated protein dependencies, neoantigens and tumor-associated antigens (Fig. 1).

The authors started by analyzing gene mutation, copy-number variations (CNVs), DNA methylation, RNA transcripts, proteomics, phosphosite data, along with an expansive analysis of several drug information databases. The identified druggable targets were classified into different tiers based on whether they were targeted by drugs approved by regulatory agencies, considered investigational, small molecules or if they were surface proteins. An analysis of mRNA-protein abundance of these druggable targets, revealed discrepancies between mRNA and protein abundance, a phenomenon expected due to posttranslational regulation, protein- protein interaction and complex formation. However, a particularly pronounced discrepancy in a group of secreted proteins was noted, emphasizing the importance of quantifying the protein abundance of druggable genes.

Even with the advancement of targeted therapy developments over the last decades, a large part of the genome and proteome remains “undruggable” in cancer.^3^ The presented analysis provides valuable information on highly overexpressed/hyperactive proteins, activating sites of kinases and phosphosites on proteins that are similar or unique to specific cancer types. The authors consolidated these findings using not only tumor tissue data but also databases containing genetic and pharmacological perturbation data from in vitro (CRISPR-Cas9 and drug response) experiments on cancer cell lines. They also compared the proteins differentially regulated due to loss-of-function (LoF) gene mutations in cancers. By integrating multi-omic analyses, they detected changes in mRNA levels, protein abundance, or protein phosphorylation associated with genomic aberrations in cancer. For example, they found that the protein levels of the topoisomerase TOP2A and its phosphorylation at site S1247 were remarkably associated with TP53 loss, a frequent event in tumors. TOP2A phosphorylation at S1247 increases its residual time on chromatin, thereby promoting transcription of cell proliferating enzymes. Additionally, their screening of cell line databases revealed that uterine cancer cell lines with TP53 loss were more sensitive to doxorubicin, a chemotherapeutic agent that inhibits TOP2A. This finding highlights how screening for TP53 loss in uterine cancer patients might potentially help in identifying adequate patient-specific therapies. Thus, associating gene mutations involved in the pathogenesis of cancer with druggable proteins and post-translational modifications is a powerful approach for designing therapeutic regimens tailored to different cancer types.

The study also combined large-scale DNA and RNA sequencing data with proteomics and phosphoproteomics information to identify probable somatic mutation-associated neoantigens across the ten cancer types. The authors identified 180 putative neoantigens from high- confidence oncogenic mutations, including genes such as *KRAS, HRAS, TP53, EGFR, ARID1A, ERBB3, CTNNB1*, and *MAPK1*. However, it should be taken under consideration that neoantigens are patient-specific, and these findings cannot be directly transferred to cohorts. Nevertheless, with the rise in the development and clinical investigation of inhibitors against KRAS mutations in cancers,^3^ it is notable that Savage et al. identified 5 promising neoepitopes candidates from KRAS mutations across lung, colon, uterine, and pancreatic cancers for drug-targeting. Using statistical analysis, the authors could further narrow down 140 tumor-associated antigens that were restrictively upregulated in cancers compared to healthy tissues. A preliminary in vitro analysis also confirmed that 22 peptides from five proteins showed strong binding affinity and immunogenicity to HLA-A*02 and could be considered for further research into immunotherapies.

The current study showcases a commendable comprehensive analysis, successfully integrating 6 omics data types (summarized at https://targets.linkedomics.org). This approach lays a strong foundation for drug repurposing and the exploration of additional treatment options. In the future it may not be limited to small molecules, but also include PROTACs, ADCs and immunotherapies. Using in silico analysis, the authors have made significant strides in predicting drug responses in cell lines based on potential drug targets. To further enhance the accuracy of drug response predictions, incorporating clinical parameters and biobanking datasets into this multi-omic analysis could be considered. Generally, the integration of clinical characteristics datasets, patient treatment history, and longitudinal follow-up data with multi- omics analyses is crucial for advancing patient-based care and precision medicine. In this context, the potential of AI-based approaches to combine multi-omics data from cells, tissues, tumors, organoids, and biobanking with detailed clinical information offers exciting opportunities. Moreover, given the complexity and heterogeneity of cancers, associating clinical biomarkers with drug interaction studies will be essential. This will not only aid in drug design and repurposing but also accelerate the entry of drugs into clinical trials. Together, these considerations are key to achieving an in-depth understanding of cancer pathogenesis and designing innovative therapeutic regimes for personalized patient care.
